# Is Endoscopic Ultrasound-Guided Hepaticogastrostomy Safe and Effective after Failed Endoscopic Retrograde Cholangiopancreatography?—A Systematic Review and Meta-Analysis

**DOI:** 10.3390/jcm13133883

**Published:** 2024-07-01

**Authors:** Saqr Alsakarneh, Mahmoud Y. Madi, Dushyant Singh Dahiya, Fouad Jaber, Yassine Kilani, Mohamed Ahmed, Azizullah Beran, Mohamed Abdallah, Omar Al Ta’ani, Anika Mittal, Laith Numan, Hemant Goyal, Mohammad Bilal, Wissam Kiwan

**Affiliations:** 1 Department of Internal Medicine, University of Missouri-Kansas City, Kansas City, MO 64108, USA; s.alsakarneh@umkc.edu (S.A.);; 2 Department of Gastroenterology and Hepatology, Saint Louis University, Saint Louis, MO 63103, USA; 3 Division of Gastroenterology, Hepatology & Motility, The University of Kansas School of Medicine, Kansas City, KS 66103, USA; 4 Department of Internal Medicine, Weill Cornell University, New York, NY 10065, USA; 5 Department of Gastroenterology and Hepatology, University of Missouri-Kansas City, Kansas City, MO 64108, USA; 6 Division of Gastroenterology and Hepatology, Indiana University School of Medicine, Indianapolis, IN 46202, USA; 7 Department of Gastroenterology and Hepatology, Cleveland Clinic Foundation, Cleveland, OH 44195, USA; 8 Department of Internal Medicine, Allegheny General Hospital, Allegheny, PA 15212, USA; 9 Division of Gastroenterology and Hepatology, Borland Groover, Jacksonville, FL 32207, USA; 10 Division of Gastroenterology and Hepatology, University of Minnesota, Minneapolis, MN 55455, USA

**Keywords:** endoscopic ultrasound-guided hepaticogastrostomy, endoscopic ultrasound, hepaticogastrostomy, endoscopic retrograde cholangiopancreatography, outcomes

## Abstract

**Background/Objectives:** Endoscopic ultrasound-guided hepaticogastrostomy (EUS-HGS) has emerged as an alternative option for biliary drainage in cases of failed endoscopic retrograde cholangiopancreatography (ERCP). Limited data exist on the safety and efficacy of EUS-HGS. In this comprehensive meta-analysis, we aim to study the safety and efficacy of EUS-HGS in cases of failed conventional ERCP. **Methods:** Embase, PubMed, and Web of Science databases were searched to include all studies that evaluated the efficacy and safety of EUS-HGS. Using the random effect model, the pooled weight-adjusted event rate estimate for clinical outcomes in each group were calculated with 95% confidence intervals (CIs). The primary outcomes were technical and clinical success rates. Secondary outcomes included overall adverse events (AEs), rates of recurrent biliary obstruction (RBO), and rates or re-intervention. **Results:** Our analysis included 70 studies, with a total of 3527 patients. The pooled technical and clinical success rates for EUS-HGS were 98.1% ([95% CI, 97.5–98.7]; I^2^ = 40%) and 98.1% ([95% CI, 97.5–98.7]; I^2^ = 40%), respectively. The pooled incidence rate of AEs with EUS-HGS was 14.9% (95% CI, 12.7–17.1), with bile leakage being the most common (2.4% [95% CI, 1.7–3.2]). The pooled incidence of RBO was 15.8% [95% CI, 12.2–19.4], with a high success rate for re-intervention (97.5% [95% CI, 94.7–100]). **Conclusions:** Our analysis showed high technical and clinical success rates of EUS-HGS, making it a feasible and effective alternative to ERCP. The ongoing development of dedicated devices and techniques is expected to make EUS-HGS more accessible and safer for patients in need of biliary drainage.

## 1. Introduction

Endoscopic retrograde cholangiopancreatography (ERCP) is the gold standard therapy for biliary obstruction for a variety of benign and malignant pancreaticobiliary disorders, with a success rate reaching up to 95% [1,2,3]. However, in cases with surgically altered anatomy or malignant duodenal obstruction, it can be very challenging and has a failure rate ranging from 5–7% in achieving biliary drainage [4]. For years, the standard alternative in this situation was limited to percutaneous transhepatic biliary drainage (PTBD). However, in recent years, endoscopic ultrasound (EUS)-guided biliary interventions have emerged as effective alternate treatment options. EUS-guided biliary drainage (EUS-BD) techniques include EUS guided rendezvous ERCP, EUS-guided choledochoduodenostomy, and EUS-guided hepaticogastrostomy (EUS-HGS) [5].

Among the EUS-BD techniques, EUS-guided hepaticogastrostomy (EUS-HGS) has gained popularity as a novel drainage technique that provides biliary decompression from the left intrahepatic duct (IHD) to the stomach [6]. This method leverages the power of endoscopic ultrasound to access the biliary system, offering distinct advantages over other conventional techniques.

Despite its overall efficacy and safety, clinicians continue to have significant concerns for bile leak and stent migration with EUS-HGSs [7,8]. Therefore, EUS-guided antegrade stenting (EUS-AGS) has emerged as a valuable alternative to EUS-HGS, particularly for patients with an inaccessible ampulla, due to its potential to establish normal bile flow [9]. Despite the fact that these techniques have been around for almost a decade, there are concerns around the safety and efficacy of these modalities. Therefore, we have conducted a systematic review and meta-analysis to evaluate the efficacy and safety of EUS-HGS and EUS-AGS in cases of unsuccessful conventional ERCP.

## 2. Methods

### 2.1. Search Strategy and Study Eligibility

Two independent reviewers (S.A. and M.M.) identified studies published before 1 October 2023, that reported on the outcomes of EUS-HGS and EUS-AGS in cases of unsuccessful conventional ERCP. We systematically searched the online MEDLINE, Embase, Cochrane, and Scopus databases using key words in different combinations: (EUS, Endoscopic Ultrasound, Ultrasound) and (Hepaticogastrostomy, biliary drainage, anterograde stenting). Additionally, according to the Preferred Reporting Items for Systematic Reviews and Meta-analyses (PRISMA), we screened the reference lists of the articles and corresponded with study investigators [10]. There was no restriction based on language as long as study outcomes were mentioned in the text. A third reviewer (O.T.) resolved any disagreement.

### 2.2. Study Inclusion and Exclusion

The inclusion criteria for studies in this analysis were as follows:

(1) Prospective or retrospective studies with a study population comprising patients with biliary obstruction.
(2) Studies involving the use of EUS-HGS or EUS-AGS as the primary intervention.
(3) Evaluation of clinical safety and efficacy as the primary outcomes.

Studies were excluded if they were case reports, case series, animal studies, editorial articles, meta-analyses, review articles, or had sample sizes smaller than 10. Studies without relevant clinical data on clinical success or adverse events were also excluded. 

### 2.3. Data Extraction and Quality Assessment

All relevant data were extracted according to a table independently predefined by S.A. and M.M. The following parameters were extracted: first author, year of publication, country, study design, patient demographics, stent type, cause of prior failed ERCP, stent patency time, technical success, functional success, and outcomes of interest. Using the Newcastle–Ottawa Scale, the methodological quality of the included cohort studies was assessed independently by two investigators (S.A. and O.T.). In the case of a discrepancy, a third independent individual (A.M) was consulted.

### 2.4. Definitions of Outcomes

The endpoint outcomes include stent patency, stent occlusion, and overall adverse events (AEs). The American Society for Gastrointestinal Endoscopy lexicon was used for the grading of the severity of procedural AEs with endoscopy [11]. Technical success of both EUS-HGS and EUS-AGS was generally defined as the successful biliary drainage as planned. Clinical success was defined as a reduction in serum bilirubin level by more than 50% at 2 to 4 weeks. Recurrent biliary obstruction was considered in case of stent migration, occlusion, or malignancy invasion of stent. Our primary outcome is technical and clinical success rate. Secondary outcomes include stent patency, stent occlusion, and adverse events.

### 2.5. Data Synthesis and Statistical Analysis

We used R version 3.2.3 (R Project for Statistical Computing) with Meta and Metaprop packages for all analyses. Using the Freeman–Tukey double arcsine transformation (FTT) method, the pooled, weight-adjusted event rate estimate for the clinical outcomes in each group was calculated using the Metaprop package. Continuity correction of 0.5 in studies with zero cell frequencies was used. Between-study heterogeneity was assessed using the Cochrane Q-statistic (I^2^), which represents the percentage of total between-study variation that cannot be attributed solely to chance. Between-study heterogeneity was rated as low if 25% < I^2^ ≤ 50%, moderate if 50% < I^2^ ≤ 75%, and high if I^2^ > 75%1. A leave-1-out meta-analysis was performed to assess the influence of the outcome by excluding each study and identifying influential studies that may contribute to heterogeneity. A subgroup analysis was performed for studies that reported the outcomes of EUS-HGS with anterograde stent. Statistical tests were 2-sided and used a significance threshold of *p* <  0.05. The assessment of publication bias was investigated by evaluation of funnel plot asymmetry and sensitivity analysis. In addition to the ethical standards of the competent institution for human subjects, this meta-analysis was conducted in compliance with the Helsinki Declaration [12].

## 3. Results

### 3.1. Literature Search and Study Characteristics

**Figure 1:**
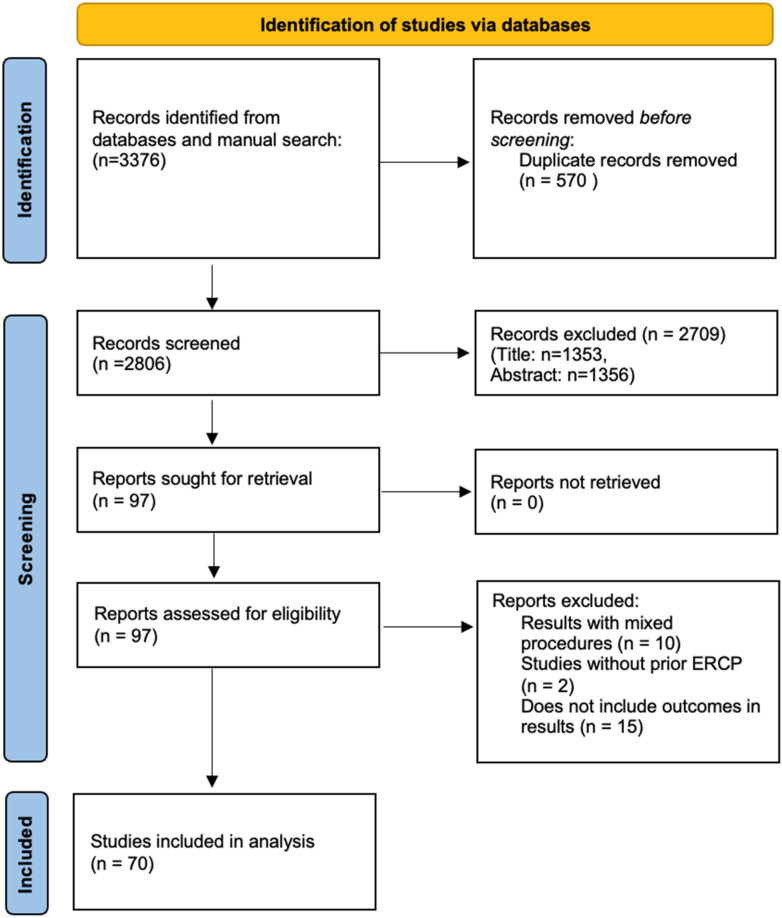
PRISMA flow chart.

A total of 3276 unique records were identified according to the above search strategy. After title and abstract screening, 70 studies with a total of 3527 patients were included in the study. PRISMA flowchart illustrates our selection process as shown in Figure 1. Supplementary Table S1 shows the baseline characteristics of the included studies and their quality analysis. Of these studies, 53 were from Asia. The study design was prospective in 19 studies, and 23 were multi-center studies. Among the included studies, 43 were of good quality, 21 were of fair quality, and 6 were of poor quality. Supplementary Table S2.

### 3.2. Baseline Characteristics of Patients and Qualitative Procedure Outcomes

Table 1 shows the baseline characteristic of patients in the studies included and procedure outcomes including: gender, age, underlying cause of obstruction, reason for prior unsuccessful ERCP, overall survival, stent patency time, median procedural time, type of stent, and location of stricture. While 61 studies included only patients with malignant obstruction, 8 included mixed malignant and benign obstruction, and 1 study included only benign obstruction. The most common cause of obstruction was pancreatic cancer. Distal bile strictures were the most common location of stricture. Metal stents were the most commonly used type. 

### 3.3. Clinical and Technical Success

**Figure 2:**
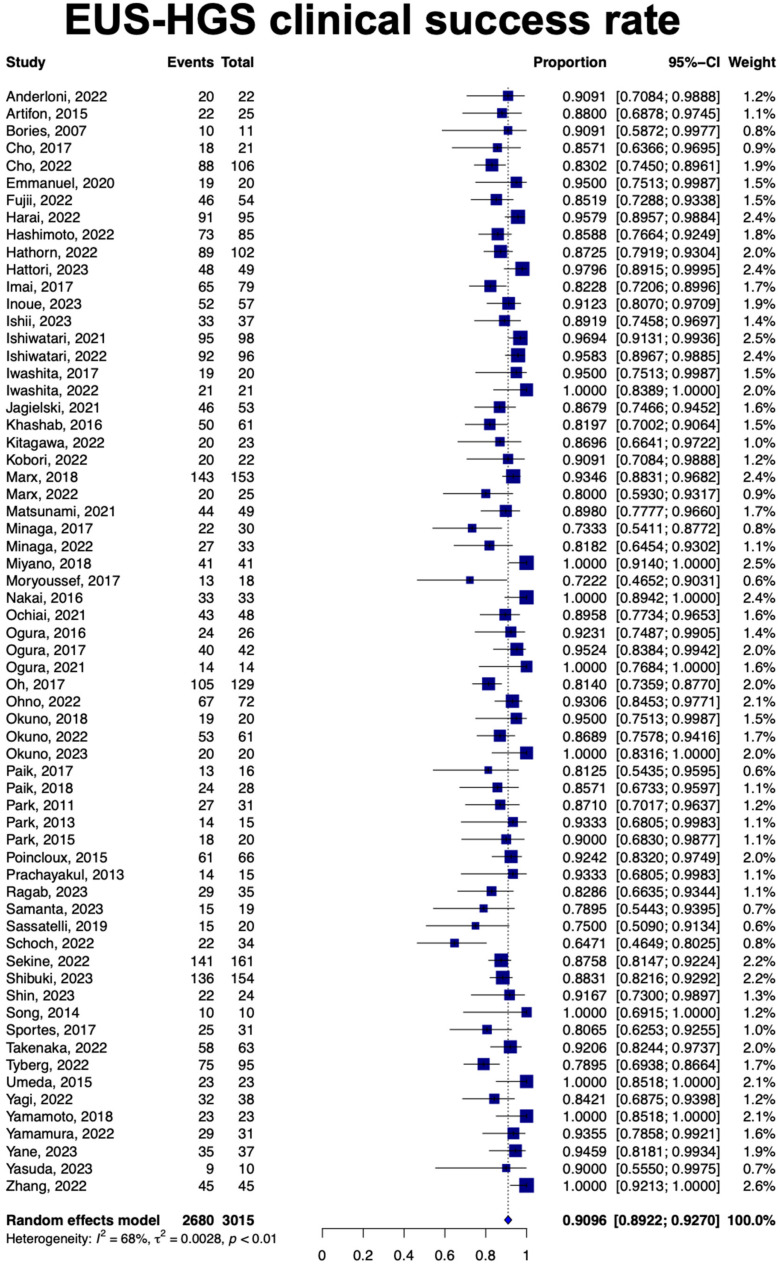
Forest plot of clinical success rat [7,13,14,15,16,17,18,19,20,21,22,23,24,25,26,27,28,29,30,31,32,33,34,35,36,37,38,39,40,41,42,43,44,45,46,47,48,49,50,51,52,53,54,55,56,57,58,59,60,61,62,63,64,65,66,67,68,69,70,71,72,73,74,75,76,77,78,79,80,81].

**Figure 3:**
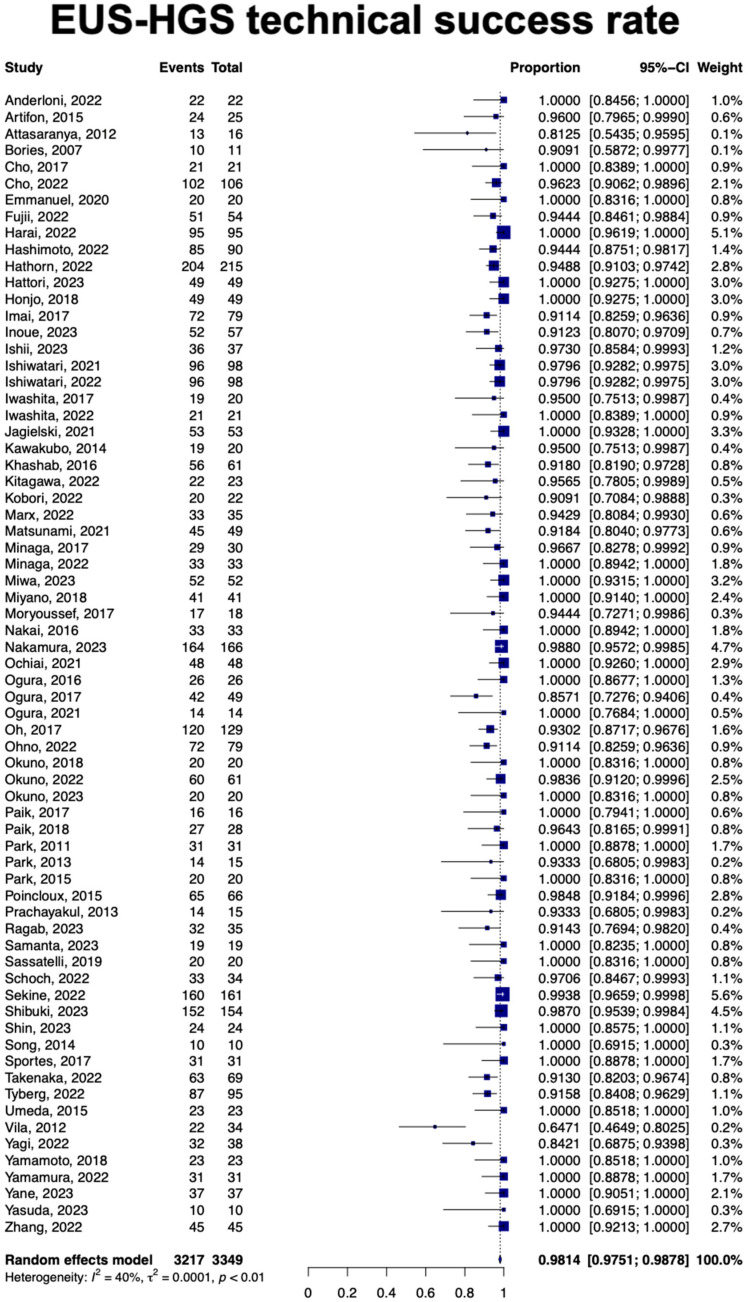
Forest plot of technical success rate [7,13,14,15,16,17,18,19,20,21,22,23,24,25,26,27,28,29,30,31,32,33,34,35,36,37,38,39,40,41,42,43,44,45,46,47,48,49,50,51,52,53,54,55,56,57,58,59,60,61,62,63,64,65,66,67,68,69,70,71,72,73,74,75,76,77,78,79,80,81].

A total of 64 studies with 3015 patients showed the pooled clinical success rate for EUS-HGS was 90.9% (95% Confidence Interval [CI], 89.2–92.7; *I*^2^ = 68%) (Figure 2). Data from 3349 patients showed a pooled technical success rate of 98.1% [95% CI, 97.5–98.7]; *I*^2^ = 40% (Figure 3). On subgroup analysis, the reported pooled clinical and technical success rates of HGAS were 95.2% [95% CI, 91.7–98.9] and 93.8% [95% CI, 89.3–98.2], respectively Table 2.

### 3.4. Overall Adverse Events

Overall, a total of 68 studies (3454 patients) reported the total number of AEs related to EUS-HGS. The pooled incidence rate of AEs with EUS-HGS was 14.9% (95% CI, 12.7–17.1; I^2^= 71%). A total of 20 studies reported the severity of AEs according to the ASGE Lexicon classification system. The results were as the following: mild: 7% [95% CI, 4.3–9.7]; moderate: 2.7 [95% CI, 1–4.5]; severe: 0.9% [95% CI, 0.1–1.7]; fatal 0.03% [95% CI, 0.0–4.6]. For the HGAS group, the pooled incidence of total AEs was 10.8% [95% CI, 6.6–15.0].

### 3.5. Individual Adverse Events

Table 1 shows the number of studies and patients and pooled incidence rate of individual AEs. The most common reported AE was bile leakage (2.4% [95% CI, 1.7–3.2]), followed by bleeding and peritonitis, with pooled incidences of 1.30% [95% CI, 0.8–1.8] and 1.27% [95% CI, 0.7–1.8], respectively. A pooled incidence of 0.5% [95% CI, 0.1–0.8] was reported for cholangitis. The pooled incidence of mortality related to the procedure was low at 0.1% [95% CI, 0.0–0.3]. The most common reported symptom was abdominal pain, with a pooled incidence of 0.13% [95% CI, 0.0–0.4]. Stent migration was reported at rate of 0.3% [95% CI, 0.1–0.6]. AEs were less frequent in HGAS group, with the most frequent reported AEs being pancreatitis with a pooled incidence of 4.7% [95% CI, 0.6–8.8]. 

### 3.6. Recurrent Biliary Obstruction (RBO) and Re-Intervention

A total of 43 studies (1919 patients) reported the rate of RBO after EUS-HGS. The pooled incidence was 15.8% [95% CI, 12.2–19.4]. However, the success rate for reintervention was high with a pooled rate of 97.5% [95% CI, 94.7–100]. 

### 3.7. Assessment of Publication Bias and Sensitivity Analysis

**Figure 4:**
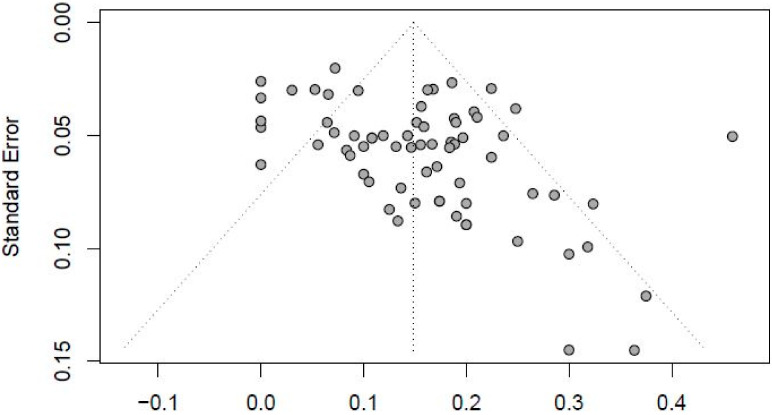
Funnel plot.

A funnel plot of included studies is shown in Figure 4, which indicates no publication bias. The symmetry of the plot around the central line suggests an even distribution of study results, implying that both positive and negative outcomes were equally likely to be published. This balanced spread of effect sizes across studies of varying sizes supports the conclusion that there is no selective publication bias affecting the meta-analysis results. Additionally, the influence of a single study on the overall meta-analysis estimate was investigated by omitting one study at a time. The omission of any study resulted in no significant difference, indicating that our results were statistically reliable. 

## 4. Discussion

In this comprehensive systematic review and metanalysis of 70 studies with 3643 patients, the pooled clinical success of EUS-HGS after unsuccessful ERCP was 90.9% and the technical success rate was 98.1%. Our analysis showed that the pooled incidence of AEs with EUS-HGS was 14.9% with bile leak being the most common AE at 2.4%. To our knowledge, this is the largest analysis including 70 studies focused on EUS-HGS. Our analysis provides valuable insights into the efficacy and safety of EUS-HGS with or without EUS-AGS when conventional ERCP fails or is not feasible.

Biliary obstruction is a challenging medical condition that can often necessitate a wide variety of interventions via endoscopic retrograde cholangiopancreatography (ERCP). When ERCP is unsuccessful, endoscopic ultrasound-guided hepaticogastrostomy (EUS-HGS) emerges as a promising alternative for biliary drainage [82]. Numerous studies have provided insights into the efficacy and safety of EUS-HGS as an option for biliary drainage [83,84,85]. The high success rates make EUS-HGS a viable alternative when conventional ERCP is not feasible, particularly in cases involving surgically altered anatomy or inaccessible papilla. Our meta-analysis showed results further supporting this expanding body of evidence, with a pooled clinical success rate of 90.9%, and a pooled technical success rate of 98.1%. Compared to endoscopic retrograde biliary drainage (ERBD) and percutaneous transhepatic biliary drainage (PTBD), EUS-HGS shows comparable success rates [86,87]. Moreover, EUS-HGS has the added advantage of being accessible in cases where ERBD is not feasible due to anatomical challenges.

Stent patency in EUS-HGS is a crucial aspect of its long-term effectiveness. Although theoretically, stent patency might be longer in EUS-HGS than in ERBD, various factors influence the duration of patency [88]. Reported stent patency durations for EUS-HGS have varied widely, ranging from 62 to 402 days. While EUS-HGS may have fewer instances of tumor ingrowth or overgrowth, it can be more susceptible to stent migration and clogging, potentially shortening stent patency. In our review which included 70 studies, there was a significant variation in the stent patency duration, ranging from 31 to 771 days with a pooled average stent patency of 155 days. The location and degree of biliary stricture, presence of gastric or duodenal obstruction, type and length of the stent used, presence of liver metastasis, and other factors all contribute to the stent patency of EUS-HGS.

In the studies included in our meta-analysis, we observed a notable variation in the reported types of stents utilized. Out of the 70 total studies, 36 (51.4%) mentioned the deployment of metal stents, while 8 studies (11.4%) specifically indicated the use of plastic stents. Additionally, 24 studies (34.3%) scrutinized the utilization of both metal and plastic stents. In contrast, 2 studies (2.9%) did not provide information regarding the type of stent used.

A recent prospective study compared stent patency between EUS-guided biliary drainage (EUS-HGS and EUS-choledochoduodenostomy) and ERBD, showing that EUS-guided drainage had significantly longer stent patency [6]. However, it is essential to consider patient survival when interpreting these results, as many patients with biliary obstruction have a short survival time. Shorter survival may reduce the likelihood of observing stent dysfunction because patients may not live long enough for the stent to fail. This distinction is crucial for understanding the actual efficacy and reliability of the stent.

EUS-HGS has been shown to produce fewer procedure-related adverse events than PTBD, making it a safer alternative [6]. The overall previously reported rate of adverse events in EUS-HGS is approximately 18%, with common adverse events including abdominal pain, self-limiting pneumoperitoneum, bile leak, cholangitis, and bleeding. However, in rare cases, serious adverse events like perforation, intraperitoneal stent migration, and mediastinitis have been reported. Notably, our analysis indicates a pooled adverse events incidence rate of 14.89%, with the most frequently encountered adverse events being bile leakage (2.4%), bleeding (1.3%), and peritonitis (1.27%). 

Compared to EUS-HGS, EUS-choledochoduodenostomy (EUS-CDS) was shown to result in less early adverse events and shorter procedure time [20]. This suggests that EUS-CDS might be a safer option for novice practitioners, while EUS-HGS should be reserved for experienced operators [89]. The learning curve for EUS-HGS is steep, and it is a technically challenging procedure. Studies suggest that achieving proficiency in EUS-HGS may require a significant number of cases, with some reports indicating that more than 33 cases may be needed to reach a plateau in the learning curve [52]. While EUS-HGS is still technically challenging, one approach to mitigate the learning curve is the conversion of PTBD to EUS-HGS for beginners [90]. This transition can offer several advantages, including the ability to identify the optimal puncture site in the intrahepatic duct via opacification through a PTBD catheter. Furthermore, it allows practitioners to become more familiar with EUS-HGS while potentially reducing the risk of adverse events, such as cholangitis or bile leak.

Despite its advantages, EUS-HGS has several limitations and challenges that need to be considered [6]. Some of these limitations include the technical complexity of the procedure, the lack of dedicated devices for EUS-HGS, the risk of serious adverse events, and the need for skilled practitioners. Additionally, there are technical challenges in draining the right liver in cases of bilateral stenosis and difficulties in patients with a non-dilated biliary system [91]. 

It is imperative to address the limitations inherent in our meta-analysis. A substantial portion of the studies incorporated in our analysis adopted a retrospective design. This approach has the potential to result in an overestimation of technical and clinical success rates, particularly when populations of patients initially assigned to EUS-HGS or EUS-AGS were subsequently transitioned to an alternative method, such as EUS-choledochoduodenostomy, and labeled as such. Furthermore, several essential parameters that might be of interest, such as the stratification of adverse events and outcomes based on common bile duct size or stent dimensions, were frequently omitted in the studies we reviewed. In recent years, various new devices, including dedicated stent systems, have been developed to make EUS-HGS more accessible [36]. These innovations aim to simplify the procedure, increase its success rate, and decrease the risk of adverse events. Continued development in this area is essential to improve the safety and feasibility of EUS-HGS.

## 5. Conclusions

EUS-HGS has emerged as a valuable alternative for biliary drainage when conventional ERCP is not feasible. The technique has shown high technical and clinical success rates and potentially longer stent patency compared to ERBD [6]. However, it is not without its challenges, including a steep learning curve, the need for skilled practitioners, and potential risks of adverse events. The ongoing development of dedicated devices and techniques is expected to address these challenges, making EUS-HGS more accessible and safer for patients in need of biliary drainage.

## Figures and Tables

**Table 1:** Comprehensive characteristics of included studies.

Study Name	Malignant/Benign Number	Male Number	Age	Underlying Cause/Diagnosis	Reason for Prior Unsuccessful ERCP (Reason and Number) for Example: 4 Due to Inability to Puncture the Bile Duct …etc.	Location of the Bile Duct Stricture (e.g., Distal: 10, Proxima: 20)	Type of Stent (e.g., PS, FCMS, MS, CMS) and Number of Each if any	Median Procedural Time in Minutes with SD	Incidence of RBO, n	Number of Successful Reinterventions(i.e., Successful Endoscopic Reintervention for RBO) Number	Median Overall Survival (95% CI), Days	Stent Patency, Mean (d) ± SD
Anderloni 2022 [13]	22 malignant	7	Mean: 66.0 ± 10.0	18 Pancreatic Cancer; 2 Cholangiocarcinoma; 1 Gallbladder Cancer; 1 Duodenal Caner	4 Infiltrated Papilla; 9 Unreachable Papila; 4 Altered Anatomy; 5 Incomplete Biliary Drainage	n/a	22 Metal	Mean: 43.3 ± 26.8	2	n/a	n/a	Mean: 10.8 ± 3.1 months
Artifon 2015 [14]	25 malignant	11	Mean: 66.25 ± 14.28	16 Pancreatic Cancer; 5 Metatstatic Adenopathy; 2 Papillary Cancer; 1 Malignent Neuroendocrine Cancer; 1 Duodenal Cancer	n/a	25 Distal	Metal	Mean: 47.8	n/a	n/a	Mean: 75.08 (5.29)	n/a
Attasaranya 2012 [15]	23 malignant	14	Mean: 58.03 ± 16.89	17 Periampullary or Pancreatic Cancer; 1 Gastric Cancer; 1 Duodenal Cancer; 1 Pancreatic Inflammatory Pseudotumor; 2 Metastatic Cancer; 3 Choledochojejunostomy Stenosis; 1 Gallstone with Cholecystitis; 1 Post-ERCP Cholecystitis; 1 CBD Stone; 1 Bile Leak; 1 Hilar Cholangiocarcinoma;1 Biloma with Postlaparoscopic Cholecystectomy	14 Failed ERCP for Biliary Cannulation; 10 Inaccessible ERCP due to Luminal Stenosis Secondary to Tumor Invasion of Gastric Antrum or Duodenum; 4 Surgically Altered Anatomy; 2 Acute Cholecystitis with Unfit Condition for Surgery; 1 Biloma	n/a	Metal	n/a	3	3/3	n/a	n/a
Bories 2007 [16]	8 malignant 3 benign	7	Mean (Range): 64 (47–80)	4 Pancreatic Cancer; 2 Hilar Cholangiocarcinoama; 1 Duodenal Cancer; 1 Gastric Cancer; 3 Benign	Failed ERCP	n/a	4 Plastic; 3 Metal	n/a	3	3/3	n/a	n/a
Cho 2017 [17]	21 malignant	16	Median (Range): 66.3 (44–82)	11 Cholangiocarcinoma; 3 Pancreatic Cancer; 3 Gallbladder Cancer; 4 Other Malignancy	14 High Grade Biliary Stricture; 6 Duodenal Obstruction; 1 Previous Operation	n/a	21 metal	Median (range): 18 (11–45)	10	5/6	Median (range): 173 (76.8–269.1)	Mean: 166.3
Cho 2022 [18]	106 malignant	68	Mean: 71.5 ± 11.2	28 Pancreatic Cancer; 42 Cholangiocarcinoma; 14 Gallbladder Cancer; 6 Ampullary Cancer; 16 Other Metastatic Disease	19 Failed ERCP; 35 Insufficient Drainiage of IHD; 32 Gastric Outlet Obstruction; 20 Surgically Altered Anatomy	41 Distal; 65 Hilar	Metal	Mean: 18.4	26	n/a	Median (IQR): 178.0 (147.7–208.3)	Median (IQR): 138.0 (70.1–205.9)
Emmanuel 2020 [19]	20 malignant	16	Mean: 71.8 ± 7.6	13 Pancreatic Cancer; 4 Periampullary Tumor; 2 Cholangiocarcinoma; 1 Metastatic Colon Cancer	16 Inaccesible Papillae; 1 Surgical Anatomy; 3 Failed Cannulation	19 Distal CBD; 1 Proximal CBD	10 Metal	Mean: 39.9 ± 1.3	1	1/1	n/a	n/a
Fujii 2022 [20]	50 malignant	28	DGW Median (IQR): 69 (56–76) SGW Median (IQR): 68 (58–72)	25 Pancreatic Cancer; 10 Biliary Cancer; 15 Other Malignancy; 4 Benign Stricture	35 Duodenal Obstruction; 14 ERCP Failure; 5 Intractable Cholangitis	34 Distal Bile Duct; 7 Perihilar Bile Duct; 13 Hepaticojejunostomy Anastomosis	11 Plastic; 42 Metal	Metal Mean (range): 47 (32–62) Plastic Mean (range): 54 (44–65)	n/a	n/a	n/a	n/a
Harai 2022 [21]	95 malignant	50	Median (IQR): 68 (58–75)	38 Pancreatic Cancer; 20 Bile Duct Cancer; 37 Other Malignancy	54 Duodenal Obstruction; 22 Surgical Anatomy; 19 Nonsuccessful ERCP	66 Distal; 29 Hilar	95 Metal	Median (IQR): 26 (17–37)	10	10/10	Median: 154 (95.0% CI 108–363)	n/a
Hashimoto 2022 [22]	85 malignant	48	Median (Range): 72 (55–90)	59 Pancreatobiliary Cancer; 26 Other Malignancy	55 Inaccessible Papilla or Ileobillary Anastomosis; 30 Accessible Papilla but Inaccessible Target Bile Duct	Distal 61; Perihilar 24	28 Plastic; 57 Metal	Median (range): 41 (11–173)	19	n/a	Median: 88 (95% CI 62.8–113.2)	Metal Range: 72–329; Plastic Range: 89–272
Hathorn 2022 [23]	130	101	Mean: 62.9 (14.7)	Cholangiocarcinoma 25, Gastric cancer 4, Pancreatic cancer 61, Ovarian cancer 1, Colorectal cancer 13, Lung cancer 5, Breast cancer 4, Ampullary carcinoma 2, Hepatocellular carcinoma 5, Pancreatic neuroendocrine tumor 2, Gallbladder 2, Vulvar cancer 1, Renal cell carcinoma 1, Duodenal adenocarcinoma 1, Malignant stricture NOS 3	n/a	n/a	Metal	n/a	n/a	n/a	n/a	n/a
Hattori 2023 [24]	37 malignant 12 benign	30	Drill Dilator Median (Range): 72 (59–92) Balloon Catheter Median (Range): 76 (48–91)	21 Pancreatic Cancer; 12 Cholangiocarcinoma; 4 Duodenal Cancer; 3 Hepaticojejunostomy Stricture; 9 Other Malignancy	27 Duodenal Obstruction; 20 Surgically Altered Anatomy; 2 Failed Biliary Cannulation	n/a	Plastic	Drill Dilator Mean: 22.7 ± 8.01; Balloon Catheter Mean: 11.1 ± 6.06	14	19/19	n/a	n/a
Honjo 2018 [25]	38 malignant	35	Mean: 68.9 ± 13.8	38 Malignant Biliary Stricture; 7 Bilioenteric Anastomosis Stricture; 4 Choledocolithiasis with Roux-en-Y	n/a	n/a	56 Plastic; 6 Metal	Mean: 21.9 ± 10.2	n/a	n/a	n/a	n/a
Imai 2017 [26]	42 malignant	24	Mean: 67.3 ± 13.9	13 Pancreatic Cancer; 18 Bile Duct Cancer; 11 Lymph Node Metastasis	n/a	n/a	Metal	Mean: 73.5 ± 29.4	n/a	n/a	68 (5–185)	Mean: 68 (5–185)
Inoue 2023 [27]	57 malignant	34	Median (IQR): 79 (69–85)	57 Pancreatic Cancer	44 Inability to Reach/Recognize the Ampulla; 13 Inability to Cannulate	57 Distal	57 Metal	Median (IQR): 25 (19–33)	16	16/16	Median: 167 (120–204)	n/a
Ishii 2023 [28]	37 malignant	22	Median (IQR): 70 (62–76)	20 Pancreatic Cancer; 6 Biliary Tract Cancer; 4 Gastric or Duodenal Cancer; 1 HCC; 6 Metastatic Lymph Node	20 Duodenal Tumor Invasion; 7 Difficult to Approach Targt; 3 Altered Anatomy; 1 Unsuccessful Biliary Cannulation; 1 History of AE from ERCPs	22 Distal; 15 Hilar	37 Metal	Median (IQR): 18 (15–24)	11	10/11	Median: 4.0 (2.0–6.1)	n/a
Ishiwatari 2021 [29]	96 malignant	58	Median (IQR): 70 (64–78)	53 Pancreatic Cancer; 15 Bliary Cancer; 28 Other Malignancy	51 MBO; 28 Surgical Anatomy	78 Distal; 18 Hilar	28 Plastic; 67 Metal	Median (IQR): 33(26–44)	n/a	n/a	n/a	n/a
Ishiwatari 2022 [30]	58 malignant	33	Median (IQR): 71 (64–78)	31 Pancreatic Cancer; 7 Biliary Cancer; 20 Others Malignancy	44 Duodenal Obstruction; 8 Surgical Anatomy; 6 Others	B2:21; B3:37	6 Plastic; 52 Metal	Median (IQR): 30 (24–39)	15	15/15	Median: 123	n/a
Iwashita 2017 [31]	20 malignant	10	Median (Range): 69 (56–92)	10 Dissemination; 5 Lymph Node Recurrent Malignancy; 4 Direction Invasion; 1 Anastomotic Recurrence	n/a	n/a	Metal	Median (range): 36.5 (10–80)	3	2/3	Median: 100.5	n/a
Iwashita 2022 [32]	21 malignant	15	Median (IQR): 71 (59.5–79)	21 Malignant Bowel Obstruction; 3 Anastomosis Stricutre; 2 Biliary Stone	n/a	n/a	Plastic or Metal	Median (IQR): 32 (27.75–49.25)	n/a	n/a	n/a	n/a
Jagielski 2021 [33]	53 malignant	38	Mean (Range): 74.66 (56–89)	19 Pancreatic Cancer; 14 Cholangiocarcinoma; 6 Gallbladder Cancer; 3 Hepatocellular Carcinoma; 6 Major Duodenal Papillary Cancer; 1 Duodenal Cancer; 2 Metastatic Colorectal Cancer; 1 Metastatic Breast Caner; 1 Metastatic Cancer of Unknown Origin	25 Duodenal Obstruction; 23 Periampullary Tumor Infiltration; 5 Failed Biliary Cannulation	n/a	Metal	Mean: 31.2 ± 15.0	3	n/a	n/a	n/a
Kawakubo 2014 [34]	20 malignant	14	Median (IQR): 72 (64–81)	11 Pancreatic Cancer; 3 Bile Duct Cancer; 1 Gallbladder Cancer; 1 Ampullary Cancer; 4 Metastatic Lymph Node; 13 Previous Biliary Drainage	14 Periamplullary Tumor Invasion; 2 Recurrent Ascending Cholangitis Due to Stent; 4 Altered GI Anatomy	n/a	Plastic and Metal	n/a	6	6/6	Median: 102 (61–262)	Mean: 51
Khashab 2016 [35]	61 malignant	38	Mean: 63.6 ± 13.8	n/a	18 Obscured Ampulla; 24 Distorted Anatomy; 14 Gastric Outlet Obstruction; 6 Others	Distal	7 Plastic; 54 Metal	Mean: 45.3 ± 34.6	12	n/a	Median: 142 (95% CI 82–256)	n/a
Kitagawa 2022 [36]	21 malignant; 2 benign	14	Mean: 73	11 Pancreatic Cancer; 1 Uterine Cancer; 4 Bile Duct Cancer; 1 Gastric Cancer; 2 Gallbladder Cancer; 1 Duodenal Cancer; 1 Intrahepatic Stone; 2 Choledocojejunal Anastomosis Stenosis	n/a	n/a	Plastic	n/a	8	4/4	n/a	n/a
Kobori 2022 [37]	20 malignant	12	Median (Range): 72 (47–90)	9 Gastric Cancer; 6 Pancreatic Cancer; 3 Bile Duct Cancer; 2 Duodenal Caner; 1 Intrahepatic Gallstone	12 Dificulty Reaching the Papilla; 7 Surgically Altered Anatomy; 3 Difficulty Cannulating the Bile Duct; 4 Presence of Cholantigis before EUS-HGS	14 Distal; 5 Hilar; 3 Anastomosis	Plastic	Median (range): 45.5 (15–90)	7	n/a	n/a	n/a
Marx 2022 [38]	205 malignant	104	Mean: 68 ± 12	64 Pancreatic Cancer; 8 Vaterian Ampuloma; 31 Cholangiocarcionma; 102 Metastasis	76 Duodenal Infiltration; 29 Altered Anatomy; 9 Failed Papillary Cannulation; 91 Hilar Stenosis with Undrained Left Liver	n/a	FCMS	n/a	47	n/a	Median: 5.3 (2.9–7.5)	Mean: 153
Marx 2022 [39]	35 malignant	28	Mean: 64 ± 11.2	n/a	n/a	n/a	Metal	n/a	10	n/a	n/a	n/a
Matsunami 2021 [40]	57 benign	28	Median (Range): 68 (7–90)	28 Bilioenteric Anastomotic Stricture; 8 Intrahepatic Biliary Stones; 15 Common Bile Duct Stones; 2 Alcoholic Chronic Pancreatitis; 1 Walled Off Necrosis; 1 Idiopathic Retroperitoneal Fibrosis; 1 Left Lobe Hepatic Injury; 1 Bile Duct Polyp	51 Surgical Anatomy; 4 Gastric Outlet Obsruction; 2 Unsuccessful ERCP	n/a	Plastic or Metal	Median (range): 22 (7–71)	n/a	n/a	n/a	n/a
Minaga 2017 [41]	30 malignant	11	Median (Range): 66 (52–87)	12 Cholangiocarcinoma; 6 Gallbladder Cancer; 5 Pancreatic Cancer; 1 Hepatocellular Carcinoma; 5 Liver Mets; 1 Lypmh Node Metastasis	4 Failed Duodenal Scope Insertion; 5 Failed Papilla Access After Duodenal Stent Insertion; 21 Failed Intrahepatic Biliary Drainage	30 Hilar	Plastic and Metal	Median (Range): 39.5 (21–68)	7	5/5	Median (range): 64 (31–314)	Mean: 62.5 (31–210)
Minaga 2022 [42]	33 malignant	22	Median (IQR): 72 (67–76)	9 Gastric Cancer; 9 Bile Duct Cancer; 8 Pancreatic Cancer; 3 Hepatocellular Cancer; 4 Other Malignancy	11 Failure of Duodenal Scope Insertion; 10 Surgically Altered Anatomy; 12 Failure of Biliary Cannulation	18 Distal; 15 proximal	33 Metal	Median (IQR): 27(20–40)	33	n/a	Median: 140 (95% CI, 70.8–209.2)	Mean: 394 days (95% CI, 85.7–702.3 days)
Miwa 2023 [43]	52 malignant	34	Median (IQR): 73 (69–80)	20 Pancreatic Cancer; 12 Biliary Cancer; 7 Colorectal Cancer; 13 Other Malignancy	27 Duodenal Obstruction; 13 Hilar Biliary Obstruction; 9 Altered Anatomy; 3 Difficult Cannulation	n/a	19 Plastic; 33 Metal	Median (IQR): 20.5 (17–30)	n/a	n/a	n/a	n/a
Miyano 2018 [44]	27 malignant	27	Extra Scope Median (Range): 70 (57–82) Intra Scope Median (Range): 75 (57–88)	13 Pancreatic Cancer; 14 Bile Duct Cancer; 14 Other Malignancy	31 Duodenal Obstruction; 10 Surgical Anatomy	B2: 3; B3: 38	Metal	n/a	na	n/a	Median: 132 (95% CI 69.3–196.3)	Extra Scope Mean: 107 days (95% CI 68.8 to 145.6); Intrascope Mean: 116 days (95% CI 57.1 to 1775.3
Moryoussef 2017 [45]	18 malignant	11	Mean: 68.8 ± 16.4	8 Pancreatic Cancer; 5 Hilar Cholangiocarcinoma; 3 Colorectal Cancer; 2 Gastric Cancer	10 Surgical Anatomy; 7 Impassible Stricture; 1 Duodenal Obstruction	18 Hilar	Metal	n/a	3	3/3	Median (range): 79 (5–390)	n/a
Nakai 2016 [46]	33 malignant	19	Median (IQR): 70 (63–77)	17 Pancreatic Cancer; 8 Biliary Tract Cancer; 2 Gastic Cancer; 2 Duodenal Cancer; 1 Hepatocellular Carcinoma; 3 Meastatic Lymph Nodes	25 Gastric Outlet Obstruction; 5 Altered Anatomy; 3 HX of Adverse ERCP	26 Distal; 7 Hilar	33 Metal	Median (IQR): 45 (30–80)	8	8/8	Median: 8.7 months (95% CI 3.1–12.6)	n/a
Nakamura 2023 [47]	166 malignant	109	Median (Range): 76 (20–94)	59 Pancreatic Cancer; 24 Cholangiocarcinoma; 16 Hepaticojejunostomy Stricture; 26 Bile Duct Stone; 14 Gastric Cancer; 8 Duodenal Cancer; 7 Gallbladder Cancer; 3 Colon Cancer; 9 Other Malignancy	84 Duodenal Invasion; 75 Surgical Altered Anatomy; 7 Failed ERCP	n/a	Plastic or Metal	Mean: 14.1 ± 8.5	n/a	n/a	n/a	n/a
Ochiai 2021 [48]	47 malignant	30	Median (IQR): 71 (50–93)	24 Pancreatic Cancer; 8 Biliary Tract Cancer; 2 Gallbladder Cancer; 4 Gastric Cancer; 2 Hepatocellular Carcionoma; 8 Other	27 Gastric Outlet Obstruction; 10 Alterd Anatomy; 10 Failed ERCP; 1 High Risk ERCP	39 Distal; 9 Hilar	47 SEMS	Median (IQR): 42(29–55)	n/a	n/a	n/a	n/a
Ogura 2016 [49]	26 malignant	13	Mean: 70 ± 8.1	21 Pancreatobililliary Cancer; 5 Others	n/a	n/a	Metal	n/a	2	n/a	Median: 113	Median: 113
Ogura 2017 [50]	49 malignant	25	Median (Range): 72 (43–96)	19 Gastric Cancer; 13 Bile Duct Cancer; 11 Pancreatic Cancer; 6 Other Malignancy	22 Duodenal Obstruction; 19 Surgical Anatomy; 8 Failed ERCP	5 Left Hepatic Bile Duct; 9 Hepatic Hilum; 3 Upper Common Bile Duct; 13 Middle Common Bile Duct; 19 Lower Common Bile Duct	Metal	n/a	7	6/6	Median: 114 (95% C.I 73.012–154.988)	Mean: 320 days (95% CI, 269.899–772.037 days)
Ogura 2021 [51]	14 malignant	8	Median (IQR): 3 (1–6)	9 Pancreatic Cancer; 3 Gastric Cancer; 2 Bile Duct Cancer	11 Duodenal Obstruction; 3 Surgically Altered Anatomy	n/a	14 Metal	Median (IQR): 7 (5–10)	1	n/a	n/a	Mean: 101 days
Oh 2016 [52]	113 malignant	81	Mean: 62.2 ± 13	n/a	52 Failure of the Guidewire Pass Across the Tight Stricture; 37 Surgically Altered Anatomy; 15 Obscured Ampulla Due to Metallic Enteral Stent; 13 Duodenal Obstruction; 10 Obscured Ampulla Due to Invasive Cancer; 2 Removal of Intrahepatic Duct Stones in Surgically Altered Anatomy	n/a	Plastic	Mean: 30.1 ± 13.1	6	5/6	n/a	Mean: 137.1 ± 243.5
Ohno 2022 [53]	72 malignant	42	Dilation + Median (Range): 69 (36–93) Dilation- Median(Range): 73 (38–92)	32 Pancreatic Cancer; 18 Biliary Tract; 8 Gastric Cancer; 14 Others Malignancy	46 Surgically Alterd Anatomy; 22 Duodenal Obstruction; 4 Unsuccessful ERCP	n/a	Dilation + 35; Dilation- 3	Dilation + Median (range): 72 (29–133); Dilation- Median (range): 44 (24–153)	1	1/1	n/a	n/a
Okuno 2018 [54]	20 malignant	12	Median: 68	9 Gastric Cancer; 1 Colon Cancer; 2 Gallbladder Cancer; 7 Pancreatic Cancer; 1 Duodenal Cancer	13 Duodenal Obstruction; 7 Altered Upper GI Anatomy	20 Distal	20 Metal	n/a	1	n/a	n/a	Mean: 87 days
Okuno 2022 [55]	55 malignant 6 benign	35	Median (Range): 68 (38–87)	28 Pancreatic Cancer; 5 Duodenal Cancer; 4 Gastric Cancer; 4 Gallbladder Cancer; 3 Colon Cancer; 3 Cholangiocellular Carcinoma; 8 Other; 6 Benign	41 Primary Drainage; 20 Salvage Drainage	7 Proximal	44 FCEMS; 16 Plastic; 1 None	Median (range): 24 (8–70)	0	n/a	n/a	n/a
Okuno 2023 [56]	18 malignant 2 benign	12	Median (Range): 70 (38–82)	6 Pancreatic Cancer; 6 Biliary Tract Cancer; 2 Gastric Cancer; 2 Hepatocellular; 1 Cholangiocellular Carcinoma; 1 Colon Cancer; 2 Anastomosis Stricture	12 Primary Drainage; 8 Salvage Drainage	n/a	Metal	Median (range): 13 (7–25)	n/a	n/a	n/a	n/a
Paik 2017 [57]	16 malignant	13	Mean: 67.6 ± 9.3	7 Cholangiocarcinoma; 2 Pancreatic Cancer; 2 Ampulla of Vater; 2 Gallbladder Cancer; 1 Hepatocellular Carcinoma; 2 Peribilary Metastasis	n/a	Distal	Metal	Mean (SD): 33.4 (20.6)	n/a	n/a	n/a	Mean: 402 days
Paik 2018 [58]	25 malignant 3 benign	20	Median (Range): 63 (29–87)	10 Cholangiocarcinoma; 5 Pancreatic Cancer; 2 Gallbladder Cancer; 2 Gastric Cancer; 1 Ampulla of Vater Malignancy; 1 Colon Cancer; 1 Duodenal Cancer; 1 Hepatocellular Carcinoma; 1 Intraductal Papillary Neoplasm of Bile Duct; 1 Lymphoma; 3 Benign	n/a	n/a	Metal	Mean: 15.6 ± 5.8	n/a	n/a	Median (range): 7.5 (5.0–12.0)	Mean: 150 (5–295) days
Park 2011 [59]	51 malignant 6 benign	35	61.7 (13)	Pancreatic cancer 12, Hilar cholangiocarcinoma 14, Ampulla of Vater cancer 5, Common bile duct cancer 3, Gallbladder cancer 2, Hepatocellular carcinoma 1, Duodenal cancer 2, Advanced gastric cancer 6, Metastatic lymph node 6	n/a	n/a	FCMS	Mean: 132	n/a	n/a	n/a	n/a
Park 2013 [7]	45 malignant	28	Mean: 64.9 ± 13	10 Pancreatic Cancer; 6 Hilar Cholangiocarcinoma; 6 Amplulla Cancer; 3 Common Bile Duct Cancer; 3 Gallbladder Cancer; 2 Hepatocellular Carcinoma; 1 Colon Cancer; 3 Lymphoma; 4 Advanced Gastric Cancer; 1 Breast Malignancy; 6 Bengin	N/A	n/a	n/a	Median: 50	n/a	n/a	n/a	n/a
Park 2015 [60]	32 malignant	20	DH Mean: 66.2 ± 11 FC Mean: 68.8 ± 13	11 Pancreatic Cancer; 13 Hilar Cholangiocarcinoma; 2 Distal Common Bile Duct Malignancy; 6 Other Malignancy	7 Surgical Anatomy; 13 High Grade Hilar Obstruction; 12 Duodenal Invastion	n/a	Metal	Median (range): 13 (10–21)	2	2/2	n/a	Mean: 121 ± 11.2 days
Poincloux 2015 [61]	98 malignant	58	Mean (Range): 70 (38–91)	51 Pancreatic Cancer; 12 Cholangiocarcinoma; 8 Ampulla Carcinoma; 3 Gallbladder Cancer; 2 Hepatocellular Carcinoma; 2 Duodenal Caner; 5 Gastric Cancer; 4 Colorectal Cancer; 3 Breast Cancer; 3 Ovarian Cancer; 2 Unknown Adenocarcinoma; 1 Pulmonary Malignancy; 1 Renal Malignancy; 3 Benign	25 Duodenal Stenosis; 7 Surgical Anatomy; 40 Periampulary Tumor Infiltration; 1 Altered Ampula Position; 1 Biliary Fistula; 27 Incomplete Draininge of High Grade Hilar Tumors	n/a	Plastic and Metal	n/a	4	n/a	n/a	n/a
Prachayakul 2013 [62]	21 malignant	10	Mean (Range): 62.8 (46–84)	9 Pancreatic Cancer; 4 Cholangiocarcinoma; 4 Gallbladder Cancer; 4 Other Malignancy	20 Obstrucive Jaundice	n/a	21 Metal	n/a	n/a	n/a	n/a	Mean: 93 days
Ragab 2023 [63]	91 malignant	59	Median (IQR): 61 (55–69)	75 Ampullary Tumor; 7 Altered Anatomy; 5 Cholangiocarcinoma; 4 Undiferentiated Common Bile Duct Malignancy	55 Inability to Achieve Deep Cannulation; 13 Duodenal Infiltration; 15 Gastric Outlet Obstruction; 8 Altered Anatomy	91 Distal	Metal, Plastic, Half to Half, Partially Covered, Fully Covered	Median (Range): 20 (15–27)	n/a	n/a	n/a	n/a
Samanta 2023 [64]	43 malignant 6 benign	23	Median (Range): 52.0 (28–76)	20 Pancreatic Cancer; 13 Gallbladder Cancer; 8 Periampullary Carcinoma; 2 Other Malignancy; 6 Benign Causes	25 Duodenal Obstruction/Inaccessible Papilla; 4 Altered Anatomy; 20 Failed ERCP	19 Hilar; 30 Distal	Metal	n/a	9	n/a	3 Month Mortality 11/49	n/a
Sassatelli 2019 [65]	36 malignant	15	Mean: 69.3 ± 12.4	25 Pancreatic Adnocarcinoma; 3 Metastatasis; 3 Cholangiocarcionma; 3 Gastric Cancer; 2 Gallbladder Cancer	13 Ampulary Obstruction by Invasive Cancer; 12 Postsurgical Anatomy; 10 Hepaticojejunostomy Stricture or Duodenal Obstruction	n/a	9 Plastic; 24 Metal	n/a	n/a	n/a	Median: 49 ± 156.7	TG-BD Mean: 72.7 ± 136.4 days TD-BD Mean: 128.5 ± 176.8 days
Schoch 2022 [66]	34 malignant	17	Median (IQR): 76 (67–83)	25 Perihilar Cholangiocarcinoma; 9 Gallbladder Cancer	22 ERCP Failure; 8 Duodenal Stricture; 2 Altered Anatomy; 2 Isolated Left Hepatic Duct Dilation	34 Perihilar	Metal	n/a	9	n/a	Median (IQR): 91 (31–263)	Mean (IQR): 145 (30–222)
Sekine 2022 [67]	144 malignant	54	B2 Mean (Range): 66.9 (32–90) B3 Mean (Range): 68.6 (32–87)	66 Pancreatic Cancer; 42 Biliary Tract; 27 Gastroduodenal Cancer; 9 Malignant Disease; 4 Bile Duct Stone; 13 Benign Disease	n/a	Distal 89; Perihilar 65; 3 Anastomosis; 1 Ampulla of Vater 1; 3 No Stenosis	114 Plastic; 47 Metal	B2 Mean (Range): 35.2 (8–110); B3 Mean (Range): 47.0 (9–187)	n/a	n/a	n/a	n/a
Shibuki 2023 [68]	154 malignant	102	Plastic Median (Range): 70 (32–85) Metal Median (Range): 69 (32–90)	62 Pancreatic Cancer; 41 Bile Duct Cancer; 28 Gastric Cancer; 21 Other Malignancy	55 Inaccessible Papilla; 33 Isolated Intrahepatic Bile Duct Obstruction; 21 Recurrent Ascenting Cholangitis; 22 Surgically Altered Anatomy; 21 Failed Biliary Cannulation	89 Distal; 63 Perihilar	109 Plastic; 43 Metal	Plastic Median (range): 30 (8–187); Metal Median (range): 41 (15–150)	47	plastic 30/35, metal 12/12	Plastic Median (range): 189 (99–270); Metal Median (range): 164 (95–281)	n/a
Shin 2023 [69]	24 malignant	7	Median (IQR): 67 (61–76)	16 Cholangiocarcinoma; 2 Pancreatic Cancer; 4 Gallbladder Cancer; 2 Ampullary Cancer	12 Failed ERCP; 7 Surgical Anatomy; 5 Gastric Outlet Obstruction	n/a	Metal	Median (IQR): 19.3 (18.4–21.2)	7	7/7	n/a	Mean: 6.7 months
Song 2014 [70]	27 malignant	13	Median (Range): 67 (29–86)	2 Pancreatic Cancer; 8 Hilar Cholangiocarcinoma; 2 Pancreatic Cancer Neuroendocrine Tumors; 2 Gallbladder Cancer; 1 Ampulla of Vater Cancer; 1 Advanced Gastric Cancer; 1 Rectal Cancer	11 Pyloric or Duodenal Obstruction; 9 High Grade Biliary Stricture; 7 Periampullary Tumor Infiltration	n/a	Metal	Median (range): 22 (14–35)	2	2	n/a	n/a
Sportes 2017 [71]	31 malignant	17	Mean: 69.2	22 Pancreatic Cancer; 5 Metatstatic Lymphadenopathy; 3 Cholangiocarcinoma; 1 Periampullary Cancer	13 Prior Surgery; 9 Duodenal Stenosis; 5 Periampullary Tumor Infiltration; 4 Impassable Stricture	n/a	Metal	n/a	2	2	Median (IQR): 71 (30–95)	n/a
Takenaka 2022 [72]	45 malignant	33	Median (IQR): 73 (65–77)	15 Pancreatic Cancer; 10 Gastric Cancer; 6 Cholangiocarcinoma; 6 Hepatocellular Carcinoma; 8 Other Malignancy	21 Failed Biliary Cannulation; 18 Surgical Anatomy; 6 Duodenal Obstruction	n/a	Plastic or Metal	Median (IQR): 15.8 (11.7–19.7)	9	9/9	n/a	n/a
Tyberg 2022 [73]	89 malignant	52	Mean: 69.9 ± 12.7	1 Ampullary Adenocarcionma; 5 Gallbladder Cancer; 19 Cholangiocarcinoma; 42 Pancreatic Cancer; 6 Colorectal Cancer; 16 Other Malignancy; 1 Choledocolithiasis	75 Obstructive Jaundice; 25 Cholangitis	n/a	8 Plastic; 82 Metal	n/a	n/a	12	n/a	n/a
Umeda 2015 [74]	15 malignant	15	Median: 77	5 Common Bile Duct Stone; 2 Ampullary Cancer; 2 Post Op Stricture; 9 Pancreatic Cancer; 1 Metastatic Lymph Nodes; 1 Bile Duct Cancer; 1 Duodenal Caner	9 Periampullary Tumor Invasion; 7 Altered Anatomy; 3 Failed Duodenal Intubation; 4 Prior ERCP Failure	n/a	Plastic	Median: 22.8	n/a	n/a	n/a	Median (Range): 4 months (0.5–9)
Vila 2012 [75]	34 malignant	n/a	n/a	n/a	n/a	n/a	n/a	n/a	n/a	n/a	n/a	n/a
Yagi 2022 [76]	27 malignant	24	Median (Range): 69 (36–84)	18 Pancreatic Cancer; 9 Billiary Cancer	n/a	26 Distal; 9 Hilar; 3 Postoperative Anastomosis	38 Metal	Median (range): 35.5 (17–80)	6	6/6	n/a	n/a
Yamamoto 2018 [77]	23 malignant	14	Median: 69 ± 12.2	11 Pancreatic Cancer; 2 Gastric Cancer; 2 Ampullary Cancer; 1 Duodenal Cancer; 1 Bile Duct Cancer; 6 Metastasis of Other Cancer	3 Failed ERCP	n/a	23 Plastic	n/a	0	n/a	Median (Range): 96 (36–656)	Mean (Range): 66 (36–462)
Yamamura 2022 [78]	31 malignant	23	Median (range): 74 (55–87)	20 Pancreatic Cancer; 9 Bile Duct Cancer; 2 Gastric Cancer	16 Duodenal Obstruction; 15 Surgically Altered Anatomy	31 Segment 3	Metal	Mean: 17.7 ± 3.76	n/a	n/a	n/a	Median: 97 (95% CI, 88–99)
Yane 2023 [79]	36 malignant	21	Median (Range): 71 (40–88)	17 Pancreatic Cancer; 10 Gastric Cancer; 2 Gallbladder Cancer; 2 Bile Duct Cancer; 5 Other Malignancy; 1 Choledocolithiasis	20 Surgical Anatomy; 10 Duodenal Obstruction; 2 Obscured Ampulla due to Invasive Cancer; 5 Segmental Cholangitis Difficult to Control with ERCP	27 Distal; 6 Hilar; 2 Choledocojejunal Anastomosis; 1 Distal plus Hilar; 1 n/a	7 Plastic; 6 Metal; 24 Both	Median (range): 35 (16–125)	0	0	n/a	n/a
Yasuda 2023 [80]	10 malignant	6	Median (Range): 66.5 (58–77)	3 Pancreatic Cancer; 5 Gastric Cancer; 1 Metastatic Colorectal Cancer; 1 Metastatic Cervical Cancer	2 Failed Biliary Cannulation	n/a	10 Metal	Median (range): 20 (15–44)	3	3/3	n/a	Mean (Range): 43 (13–215)
Zhang 2022 [81]	24 malignant	4	Mean: 69.3 ± 6.8	n/a	19 Surgically Altered Anatomy; 5 Gastrointestinal Obstruction	n/a	24 Plastic	n/a	1	1/1	n/a	Mean: 141.0 ± 73.6

**Table 2:** Details of clinical and technical success rates and adverse events associated with EUS-HGS and HGAS.

	EUS-HGS	HGAS
**Success rate**		
Clinical success	90.9 (89.2–92.7)	95.2 (91.7–98.9)
Technical success	98.1 (97.5–98.7)	93.8 (89.3–98.2)
**Adverse events**		
Overall adverse events	14.9 (12.7–17)	10.8 (6.6–15.0)
Bile leakage	2.4 (1.7–3.2)	0.1 (0.0–1.1)
Bleeding	1.3 (0.8–1.8)	1.6 (0.5–2.7)
Peritonitis	1.27 (0.7–1.8)	1.1 (0.6–1.6)
Cholangitis	0.5 (0.1–0.8	0.5 (0–2.5)
Mortality	0.1 (0.0–0.3)	0 (0.0–0.5)
Abdominal pain	0.13 (0.0–0.4)	0 (0.0–1.2)
Stent migration	0.3 (0.1–0.6)	0 (0.0–1.5)
Sepsis	0.5 (0.1–0.8)	0 (0.0–1.3)
Pneumoperitoneum	0.1 (0.0–0.4)	0 (0.0–1.0)
Perforation	0.1 (0.0–0.3)	0 (0.0–1.1)
Cholecystitis	0.1 (0.0–0.6)	0 (0.0–0.9)
**ASGE lexicon classification of adverse events severity**		
Mild	7 (4.3–9.7)	NA
Moderate	2.7 (1–4.5)	NA
Severe	0.9 (0.1–1.7)	NA
Fatal	0.03 (0.0–4.6)	NA
**Recurrent obstruction and reintervention success rate**		
RBO	15.8 (12.2–19.4)	NA
Reintervention success	97.5 (94.7–100)	NA

Values are percentages (%) with the corresponding (95% confidence interval) EUS-HGS: Endoscopic Ultrasound Hepaticogastostomy. HGAS: EUS-guided antegrade stenting.

## Supplementary Materials

The following supporting information can be downloaded at: https://www.mdpi.com/article/10.3390/jcm13133883/s1, Table S1: Baseline characteristics of the included studies and their quality level; Table S2: Quality assessment of included studies using Newcastle-Ottawa Scale.
