# Supplementary material for: Is Endoscopic Ultrasound-Guided Hepaticogastrostomy Safe and Effective after Failed Endoscopic Retrograde Cholangiopancreatography?—A Systematic Review and Meta-Analysis
Source: J Clin Med. 2024 Jul 1;13(13):3883. doi: 10.3390/jcm13133883 (PMC11242375; doi:10.3390/jcm13133883)
Supplement: Supplementary file 1 [file jcm-13-03883-s001.zip › jcm-3009929-supplementary.pdf]

SUPPLIMENTARY MATERIAL

Table S1: Baseline characteristics of the included studies and their quality level

Study name, year	Country	Center	Design	Quality
Anderloni, 2022	Italy	Single	Prospective	Good
Artifon, 2015	Brazil	Single	Prospective	Good
Attasaranya, 2012	Thailand	Single	Retrospective	Fair
Bories, 2007	France	Single	Retrospective	Poor
Cho, 2017	South Korea	Single	Prospective	Good
Cho, 2022	South Korea	Multiple	Prospective	Good
Emmanuel, 2020	Malaysia	Single	Retrospective	Poor
Fujii, 2022	Japan	Single	Retrospective	Good
Harai, 2022	Japan	Single	Retrospective	Fair
Hashimoto, 2022	Japan	Multiple	Retrospective	Good
Hathorn, 2022	United States	Single	Retrospective	Poor
Hattori, 2023	Japan	Single	Retrospective	Fair
Honjo, 2018	Japan	Single	Retrospective	Fair
Imai, 2017	Japan	Single	Retrospective	Fair
Inoue, 2023	Japan	Single	Retrospective	Good
Ishii, 2023	Japan	Multiple	Retrospective	Good
Ishiwatari, 2021	Japan	Single	Retrospective	Good
Ishiwatari, 2022	Japan	Single	Retrospective	Good
Iwashita, 2017	Japan	Single	Prospective	Fair
Iwashita, 2022	Japan	Multiple	Retrospective	Poor

Jagielski, 2021	Poland	Single	Prospective	Good
Kawakubo, 2014	Japan	Multiple	Retrospective	Good
Khashab, 2016	Multiple (US, Europe, Asia)	Multiple	Retrospective	Good
Kitagawa, 2022	Japan	Multiple	Retrospective	Good
Kobori, 2022	Japan	Multiple	Retrospective	Good
Marx, 2022	France	Multiple	Prospective	Good
Marx, 2022	France	Multiple	Prospective	Good
Matsunami, 2021	Japan	Single	Retrospective	Good
Minaga, 2017	Japan	Single	Retrospective	Good
Minaga, 2022	Japan	Multiple	Retrospective	Fair
Miwa, 2023	Japan	Single	Retrospective	Good
Miyano, 2018	Japan	Single	Retrospective	Good
Moryoussef, 2017	France	Single	Prospective	Good
Nakai, 2016	Japan	Multiple	Retrospective	Good
Nakamura, 2023	Japan	Single	Retrospective	Fair
Ochiai, 2021	Japan	Single	Retrospective	Good
Ogura, 2016	Japan	Single	Retrospective	Fair
Ogura, 2017	Japan	Multiple	Prospective	Fair
Ogura, 2021	Japan	Single	Retrospective	Fair
Oh, 2017	Korea	Single	Prospective	Fair
Ohno, 2022	Japan	Multiple	Retrospective	Fair
Okuno, 2018	Japan	Single	Prospective	Fair
Okuno, 2022	Japan	Single	Retrospective	Good
Okuno, 2023	Japan	Single	Retrospective	Good
Paik, 2017	Korea and Japan	Multiple	Retrospective	Good
Paik, 2018	South Korea	Single	Prospective	Fair

Park, 2011	Korea	Single	Prospective	Good
Park, 2013	Korea	Single	Prospective	Good
Park, 2015	Republic of Korea	Multiple	Prospective	Good
Poincloux, 2015	France	Single	Retrospective	Good
Prachayakul, 2013	Thailand	Single	Retrospective	Fair
Ragab, 2023	Egypt	Multiple	Prospective	Fair
Samanta, 2023	India	Multiple	Retrospective	Fair
Sassatelli, 2019	Italy	Single	Retrospective	Good
Schoch, 2022	France	Single	Retrospective	Fair
Sekine, 2022	Japan	Multiple	Retrospective	Fair
Shibuki, 2023	Japan	Multiple	Retrospective	Good
Shin, 2023	France	Single	Prospective	Good
Song, 2014	South Korea	Single	Prospective	Good
Sportes, 2017	France	Multiple	Retrospective	Good
Takenaka, 2022	Japan	Single	Retrospective	Poor
Tyberg, 2022	Multiple	Multiple	Retrospective	Good
Umeda, 2015	Japan	Single	Retrospective	Good
Vila, 2012	Spain	Multicenter	Retrospective	Good
Yagi, 2022	Japan	Single	Retrospective	Poor
Yamamoto, 2018	Japan	Single	Retrospective	Fair
Yamamuru, 2022	Japan	Single	Retrospective	Good
Yane, 2023	Japan	Single	Retrospective	Good
Yasuda, 2023	Japan	Single	Retrospective	Good
Zhang, 2022	China	Single	Retrospective	Good

Table S2: Quality assessment of included studies using Newcastle-Ottawa Scale

First author and year	Selection				Comparability	Outcome			Quality	
	Representativeness of the exposed cohort	Selection of the nonexposed cohort	Ascertainment of exposure	Demonstration that outcome of interest was not present at start of study	Comparability of cohorts based on the design or analysis	Assessment of outcome	Was follow-up long enough for outcomes to occur	Adequacy of follow up of cohorts	Score	Quality
Anderloni, 2022	*		*		**	*	*	*	7	Good
Artifon, 2015	*	*	*	*	**	*	*	*	9	Good
Attasaranya, 2012			*		*	*	*	*	5	Fair
Bories, 2007			*		*	*		*	4	Poor
Cho, 2017	*		*		**	*	*	*	7	Good
Cho, 2022	*		*	*	**	*	*	*	8	Good
Emmanuel, 2020			*		**	*		*	4	Poor
Fujii, 2022	*		*		**	*	*	*	7	Good
Harai, 2022			*		**	*	*	*	6	Fair
Hashimoto, 2022	*	*	*	*	**	*	*	*	9	Good

Hathorn, 2022			*		*	*		*	4	Poor
Hattori, 2023	*			*	**	*		*	6	Fair
Honjo, 2018	*		*		**	*		*	6	Fair
Imai, 2017			*		**	*	*	*	6	Fair
Inoue, 2023	*		*	*	**	*	*	*	8	Good
Ishii, 2023			*	*	**	*	*	*	7	Good
Ishiwatari, 2021	*		*	*	**	*	*	*	8	Good
Ishiwatari, 2022	*		*		**	*	*	*	7	Good
Iwashita, 2017			*			*	*	*	5	Fair
Iwashita, 2022			*		*	*		*	4	Poor
Jagielski, 2021		*	*	*	**	*	*	*	8	Good
Kawakubo, 2014			*	*	**	*	*	*	7	Good
Khashab, 2016	*		*	*	**	*	*	*	8	Good
Kitagawa, 2022			*	*	**	*	*	*	7	Good
Kobori, 2022	*		*	*	**	*	*		7	Good
Marx, 2022	*		*	*	**	*	*	*	8	Good
Marx, 2022	*		*	*	**	*	*	*	8	Good

Matsunami, 2021	*		*	*	**	*	*	*	8	Good
Minaga, 2017	*		*		**	*	*	*	7	Good
Minaga, 2022	*		*		**	*	*		6	Fair
Miwa, 2023	*		*		**	*	*	*	7	Good
Miyano, 2018	*		*	*	**	*	*	*	8	Good
Moryoussef, 2017	*		*		**	*	*	*	7	Good
Nakai, 2016	*		*		**	*	*	*	7	Good
Nakamura, 2023			*		**		*	*	5	Fair
Ochiai, 2021	*		*		**	*	*	*	7	Good
Ogura, 2016	*		*		*	*	*	*	6	Fair
Ogura, 2017			*		*	*	*	*	5	Fair
Ogura, 2021			*	*	**	*			5	Fair
Oh, 2017			*		*	*	*	*	5	Fair
Ohno, 2022			*		**	*	*		5	Fair
Okuno, 2018			*		**	*	*	*	6	Fair
Okuno, 2022	*		*		**	*	*	*	7	Good
Okuno, 2023	*		*		**	*	*	*	7	Good
Paik, 2017			*	*	**	*	*	*	7	Good
Paik, 2018			*		**	*	*	*	6	Fair
Park, 2011	*	*	*		**	*	*	*	8	Good
Park, 2013	*	*	*	*	**	*	*	*	9	Good

Park, 2015	*	*	*	*	**	*	*	*	9	Good
Poincloux, 2015	*		*		**	*	*	*	7	Good
Prachayakul , 2013	*		*	*				*	5	Fair
Ragab, 2023			*	*	*	*	*	*	6	Fair
Samanta, 2023			*		**	*		*	5	Fair
Sassatelli, 2019	*		*		**	*	*	*	7	Good
Schoch, 2022			*		**	*	*		5	Fair
Sekine, 2022	*		*		**	*		*	6	Fair
Shibuki, 2023	*		*		**	*	*	*	7	Good
Shin, 2023	*		*		**	*	*	*	7	Good
Song, 2014	*		*		**	*	*	*	7	Good
Sportes, 2017	*		*		**	*	*	*	7	Good
Takenaka, 2022			*		*	*		*	4	Poor
Tyberg, 2022	*		*	*	**	*	*	*	8	Good
Umeda, 2015	*		*	*	**	*	*	*	8	Good
Vila, 2012	*		*		**	*	*	*	7	Good
Yagi, 2022			*		*	*		*	4	Poor
Yamamoto, 2018			*		*	*	*	*	5	Fair

Yamamuru, 2022			*	*	**	*	*	*	7	Good
Yane, 2023	*		*	*	**	*		*	7	Good
Yasuda, 2023	*		*		**	*	*	*	7	Good
Zhang, 2022	*		*	*	**	*	*	*	8	Good
